# Tracheal colonization factor A (TcfA) is a biomarker for rapid and specific detection of *Bordetella pertussis*

**DOI:** 10.1038/s41598-020-72092-6

**Published:** 2020-09-14

**Authors:** Amanda R. Burnham-Marusich, Ryan K. Olsen, Jacqueline Scarbrough, Alexander Kvam, Wei Yang, Lindsey Zimmerman, James J. Dunn, Tod Merkel, Thomas R. Kozel

**Affiliations:** 1 DxDiscovery, Reno, NV USA; 2 Department of Microbiology and Immunology, University of Nevada, Reno School of Medicine, Reno, NV USA; 3 Department of Community Health Sciences, University of Nevada, Reno, Reno, NV USA; 4 Center for Biological Evaluation and Research, Food and Drug Administration, Silver Spring, MD USA; 5 Texas Children’s Hospital, Houston, TX USA; 6 Present Address: Department of Chemistry, University of Nevada, Reno, Reno, NV USA; 7 Present Address: Department of Molecular, Genetic and Population Health Sciences, University of Edinburgh, Edinburgh, Scotland, UK; 8 Present Address: Charles River Laboratories, Reno, NV USA

**Keywords:** Diagnostic markers, Bacterial infection, Bacterial infection, Infectious-disease diagnostics, Clinical microbiology

## Abstract

Pertussis is a highly contagious disease for which prompt, point-of-care diagnosis remains an unmet clinical need. Results from conventional test modalities (nucleic acid detection, serology, and culture) take hours to days. To overcome this challenge, we identified a new biomarker (tracheal colonization factor A, TcfA) for detection of *Bordetella pertussis* infection by lateral flow immunoassay (LFIA). We developed a library of 28 epitope-mapped monoclonal antibodies against TcfA and incorporated three antibodies into a LFIA. The LFIA did not cross-react with common bacterial or fungal organisms, but did react with nine distinct *B. pertussis* strains. The minimal linear epitope sequences targeted by the LFIA were conserved in 98% of 954 *B. pertussis* isolates collected across 12 countries from 1949–2017. The LFIA’s limit of detection was 3.0 × 10^5^ CFU/mL with *B. pertussis* cells in buffer, 6.2 × 10^5^ CFU/mL with nasopharyngeal washes from a non-human primate model, and 2.3 ng/mL with recombinant TcfA. The LFIA reacted with patient nasopharyngeal swab specimens containing as few as 1.8 × 10^6^ *B. pertussis* genomes/mL and showed no false-positives. Rapid (< 20 min) LFIA detection of TcfA as a biomarker for *B. pertussis* infection is feasible and may facilitate early detection of pertussis.

## Introduction

Pertussis (whooping cough) is a serious respiratory disease caused by *Bordetella pertussis*. It is airborne^1^, highly contagious^2,3^, and responsible for an annual 18.4 million illnesses and 254,000 deaths worldwide^4^. Despite high vaccine coverage, pertussis has persisted in the United States with recent peaks of approximately 48,000 reported cases in 2012 and 33,000 cases in 2014^5,6^. Moreover, reported cases likely underestimate the true number of infections^7,8^.

Diagnostics for pertussis include nucleic acid amplification, culture, and serology. The most appropriate test to use for a given patient depends on several factors, including the elapsed time since symptom onset and patient vaccination status. Ideally, clinical, epidemiological, and laboratory data are used together. Serologic testing is recommended for patients whose symptoms began several weeks prior. However, serology cannot be performed on patients who have been recently vaccinated, which is problematic for infants and children. For patients with early-stage disease, culture and nucleic acid amplification are recommended. Culture has high specificity, but its clinical utility is limited by its slow turnaround time (5–7 days for results) and low sensitivity^9^. Nucleic acid amplification tests include a number of commercially-available, FDA-cleared assays^10,11^ as well as lab developed real-time PCR (qPCR) tests^12^. Current molecular tests typically have high sensitivity and specificity, but are also slow (sample to result times greater than 1 h), require specialized equipment and user expertise, and can be expensive. For almost all molecular pertussis testing, the typical real-world turnaround time from sample collection to result is 1–2 days.

Prompt diagnosis is critical for minimizing the extent and economic burden of pertussis outbreaks. Antibiotic treatment decreases patients’ infectious period and limits transmission^3^. Moreover, once patients are diagnosed, high-risk close contacts can receive prophylactic antibiotics^13^. For infants, early diagnosis could also save lives. Infants have the highest risk of mortality and severe neurological complications^14^, yet infants require more doctor visits to reach a pertussis diagnosis than older patients^15^.

Rapid, point-of-care diagnosis of pertussis would enable the immediate initiation of appropriate antibiotic therapy and outbreak containment measures. Consequently, the present study was designed to (i) identify a biomarker for *B. pertussis* infection amenable to point-of-care detection by rapid lateral flow immunoassay (LFIA) technology, (ii) develop a library of high-quality monoclonal antibodies (MAbs) against the candidate biomarker, (iii) incorporate the MAbs into a LFIA, and (iv) evaluate the feasibility of detecting *B. pertussis* infection by LFIA in less than 20 min from nasopharyngeal specimens.

## Results

### Biomarker selection and epitope validation with polyclonal antibodies

Candidate *B. pertussis* biomarkers were selected based on their high abundance^16,17^, known immunogenicity during human infection^9,18^ or animal immunization^17,19–21^, and secreted or surface localization^17,20,22–24^. Candidates that were known virulence determinants or cytotoxic factors^9^ were prioritized to increase the probability of a diagnostic’s long-term utility due to selective pressure against loss of expression. Candidates were also required to have multiple, spatially separated regions with high predicted antigenicity, high conservation across *B. pertussis* strains, and low homology to other microorganisms reported in nasopharyngeal specimens^25–31^. Ultimately, we selected five proteins that met the above criteria: tracheal colonization factor A (TcfA), adenylate cyclase toxin (ACT), filamentous hemagglutinin (FHA), pertussis toxin subunit 1 (PTXS1), and autotransporter (Vag8). For each protein, we designed two to five peptide immunogens and used them to generate rabbit pAbs. The resulting 20 affinity-purified rabbit pAbs were evaluated for their performance in ELISA (summarized in Table 1). The TcfA pAbs produced the most sensitive antigen-capture ELISA for formaldehyde-inactivated *B. pertussis* cells. Thus, we focused our MAb development efforts on TcfA as a biomarker and on the three TcfA peptides that yielded the most highly reactive TcfA pAbs as target epitopes.

**Table 1:** Reactivity of purified pAbs raised against predicted epitopes of TcfA, ACT, FHA, PTXS1, and Vag8.

	Target				
	TcfA	ACT	FHA	PTXS1	Vag8
Number of peptide immunogens for each protein	4	4	5	5	2
Number of pAbs reactive with *B. pertussis* protein^a^	4	4	5	2	2
Number of pAb pairs capable of antigen-capture ELISA^b^	8	5	1	0	0
LOD with *B. pertussis* cells (10^6^ CFU/mL)^c^	4.1	> 3900	> 3900	NA	NA

All pAbs were affinity purified using each cognate peptide in the solid phase.

^a^pAb reactivity determined by ELISA using (i) *B. pertussis* cells, (ii) cell lysate, or (iii) purified protein in the solid phase as the detected antigen.

^b^pAb function in antigen-capture ELISA determined by assessing all possible pairwise permutations of epitope-specific pAbs in the capture or detector modes.

^c^LOD was defined as the concentration of formaldehyde-inactivated *B. pertussis* cells that generated signal equal to 3 × background. The average LOD was calculated by assessing the best pAb pair on three days, each day in duplicate. Directly measured OD_600_ values were converted to CFU/mL based on OD_600_ measurements and quantitative cultures performed with live *B. pertussis.*

### Monoclonal antibody development and epitope mapping

Mice were immunized with various combinations of formaldehyde-inactivated *B. pertussis* cells, tag-free recombinant TcfA (rTcfA), and/or TcfA peptides conjugated to KLH. Hybridomas were generated from the splenocytes. The resulting MAbs were purified and characterized for broad epitope reactivity (i.e*.* reactivity with BSA conjugates containing amino acids 140-160, 288-304, or 305-323 of TcfA) (Supplementary Fig. S1).

**Figure 1:**
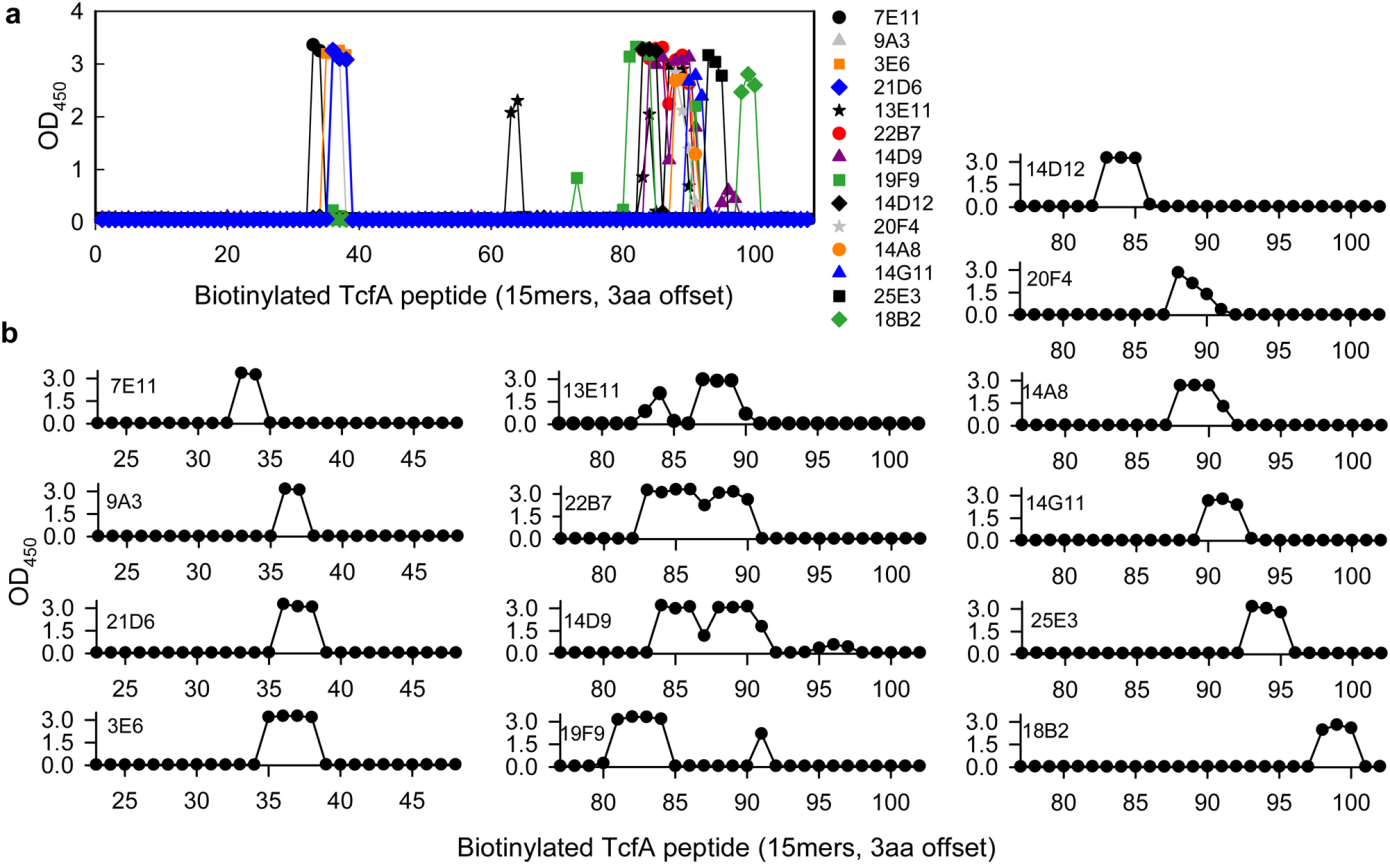
Identification of MAb minimal linear epitopes by indirect ELISA with a tiled library of biotinylated TcfA peptides. Purified MAbs were evaluated by indirect ELISA with streptavidin plates loaded with biotinylated TcfA peptides (15mers, offset by three amino acids). (**A**) Representative data from one MAb per epitope group across all 108 peptides in the library is shown. (**B**) Close-up showing data from only peptides 23-48 or peptides 77-102 for the representative MAbs.

To finely map the minimal linear epitopes of the MAbs, a tiled library of biotinylated TcfA peptides was probed by ELISA. Minimal linear epitopes were defined as the minimal overlapping peptide sequence for wells with an OD_450_ greater than 0.75 (and in a series of two or more such adjacent wells). The 28 MAbs belonged to 14 minimal linear epitope groups (Fig. 1 and diagramed in Supplementary Fig. S2).

### MAb performance in LFIA

**Figure 2:**
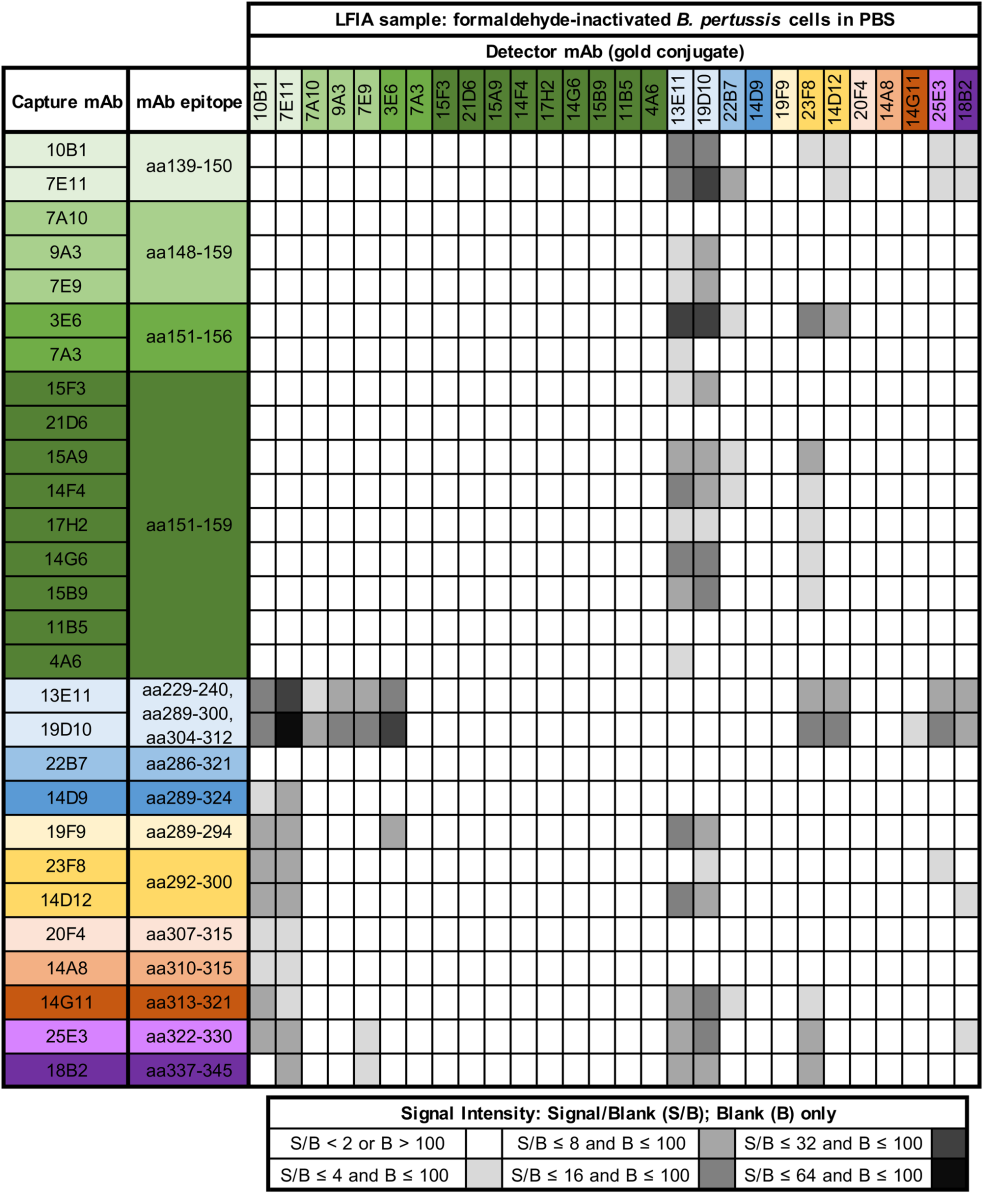
Heat map of pairwise MAb screening to identify LFIA-compatible MAb pairs and MAb pair orientations. Each MAb was evaluated for its performance as an LFIA test line capture MAb (rows) and as a gold conjugate detector MAb (columns) with formaldehyde-inactivated *B. pertussis* cells in PBS at an OD_600_ of 2 and with PBS alone. All LFIA permutations that produced more signal with PBS containing *B. pertussis* cells than with PBS alone (as determined by visual inspection) were quantified with an electronic reader. Shaded boxes within the heat map indicate the ratio of signal to blank (average of two replicates). MAbs are ordered by their epitope, with the most N-terminal binding MAb appearing at the top (column) and left (row). MAb labels are colored according to their epitope; each of the 14 minimal linear epitopes are a different combination of color and shade. In addition, MAbs that bind aa140-160 of TcfA are light to dark green, MAbs that bind both aa288-304 and aa305-323 are light to dark blue, MAbs that bind aa288-304 are light to dark yellow, MAbs that bind aa305-323 are light to dark orange, and MAbs that bind none of these regions are light to dark purple.

To identify functional MAb pairings for LFIA, we screened each MAb for performance as a test line MAb and as a gold conjugate MAb (Fig. 2). Of 784 MAb pair permutations evaluated by LFIA, we identified 101 functional permutations that showed a twofold or greater increase in signal with formaldehyde-inactivated *B. pertussis* cells in PBS *vs.* PBS alone and that showed little to no absolute background (less than 100 units of test line signal with PBS alone) (Fig. 2).

**Figure 3:**
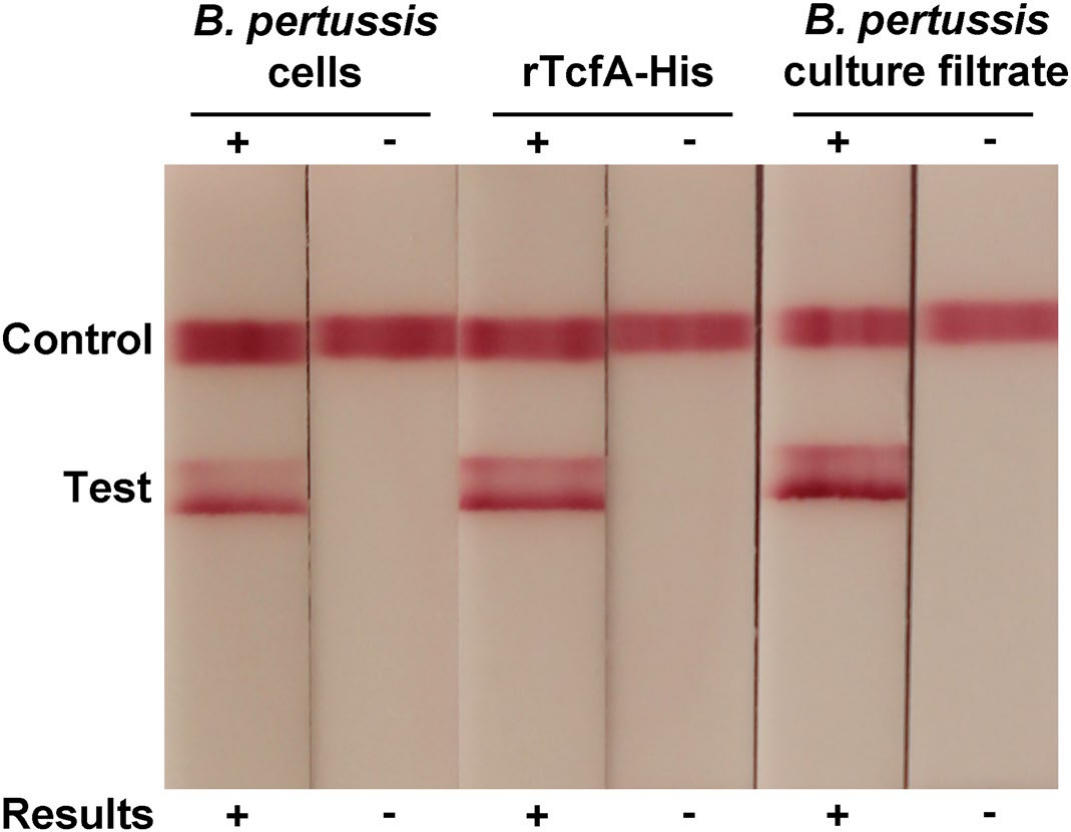
LFIA constructed with MAbs 13E11 and 14D12 at the capture line and MAb 10B1 as the gold conjugate detector reacts with multiple *B. pertussis* antigen preparations. From left to right, LFIAs were tested with the following samples: (i) formaldehyde-inactivated *B. pertussis* cells in PBS (OD_600_ of 0.5) lysed for 5 min with 0.25% SDS, ((ii) 0.25% SDS in PBS, (iii) rTcfA-His (250 ng/mL) in 0.25% SDS in PBS, (iv) 0.25% SDS in PBS, (v) 0.2 µm-filtered supernatant from a Stainer-Scholte *B. pertussis* culture (diluted 1:10 in 0.25% SDS in PBS), or (vi) uninoculated Stainer-Scholte medium (diluted 1:10 in 0.25% SDS in PBS).

The most sensitive permutations were advanced for additional testing with (i) formaldehyde-inactivated *B. pertussis* cells lysed in SDS, (ii) unfixed 0.2 µm-filtered supernatant of *B. pertussis* liquid cultures, (iii) rTcfA-His, and (iv) viable *B. pertussis* cells in PBS (data not shown). LFIAs containing cocktails of three MAbs (two as gold conjugates and one at the test line or vice versa) were also evaluated for a subset of the highest performing MAbs (data not shown). Ultimately, the LFIA containing MAb 10B1 as gold conjugate and MAbs 13E11 and 14D12 at the test line was chosen for further study based on its consistently strong reactivity with a diversity of *B. pertussis* antigen preparations and lack of detectable background (Fig. 3).

### LFIA analytical specificity and inclusivity

The chosen LFIA was evaluated for analytical specificity with titered vials of viable bacteria and fungi. Samples were tested in triplicate at 3.3 × 10^7^ CFU/mL; *B. pertussis* (Tohama I) was included as a positive control. The LFIA showed no cross-reactivity with any of the 40 species tested, including two strains each of *B. parapertussis* and *B. bronchiseptica,* and one strain of *B. holmesii* (Supplementary Table S1).

Inclusivity of the LFIA was evaluated with formaldehyde-inactivated *B. pertussis* cells at an OD_600_ of 0.1 (equivalent to 4.8 × 10^7^ CFU/mL) across 10 LFIA replicates per strain. All strains isolated from 1963 onwards reacted with the LFIA (n = 8, Tohama I, 165, H735, H792, E431, CNCTC Hp 12/63, H973, and A639) (Supplementary Fig. S3). The two strains that did not react were 18323 and 5374(3747), which were isolated prior to 1947 and 1957, respectively^32,33^. Strain 18323 carries the *tcfA1* allele, which contains an extra 25 amino acids relative to the *tcfA2* allele carried by the Tohama I strain^32,34^. Alignment of the two alleles along with the minimal linear epitopes for the MAbs in the LFIA are diagramed in Supplementary Fig. S4.

To further evaluate how inclusive the LFIA would be with recent clinical isolates, we analyzed the published genomic sequences of 170 *B. pertussis* isolates selected by the Centers for Disease Control as representative of *B. pertussis* diversity in the U.S. from 2000 to 2013^35^. All 170 isolates contained sequences encoding the same protein as the *tcfA2* allele, which is reactive with the pertussis LFIA. Broadening our geographic focus, we reviewed 784 other *B. pertussis* isolates for which TcfA sequence information was available^34,36–40^. Only 19 of these isolates were from the U.S.^34,39^. The remaining 765 were from Poland, the Netherlands, China, the United Kingdom, Finland, Sweden, France, Germany, Japan, Italy, and Belgium (listed in descending order based on the number of isolates sourced from each country)^34,36–40^. These isolates were collected between 1949 and 2017^34,36–40^. Of these 784 isolates, 90% (n = 706 isolates) harbored the *tcfA2* allele. In addition, 59 of the 784 isolates carried the *tcfA3* or *tcfA4* alleles, which contain a single amino acid substitution ≥ 23 amino acids away from the nearest minimal linear epitope sequence of any of the three MAbs in the LFIA (i.e. position 176 for *tcfA3* and 174 for *tcfA4*)^34,36^ (Fig. 1 and Supplementary Fig. S2). As a consequence, 765 of the 784 isolates (98%) are predicted to be reactive with the three MAbs in the LFIA. The remaining 19 isolates comprise *tcfA* deletions (n = 6), frameshifts (*tcfA5*, n = 7; *tcfA9*, n = 1) or alternative alleles (*tcfA1*, n = 3; *tcfA6*, n = 2)^34,36–39^.

### LFIA analytical sensitivity with antigen in buffer and baboon nasopharyngeal specimens

**Figure 4:**
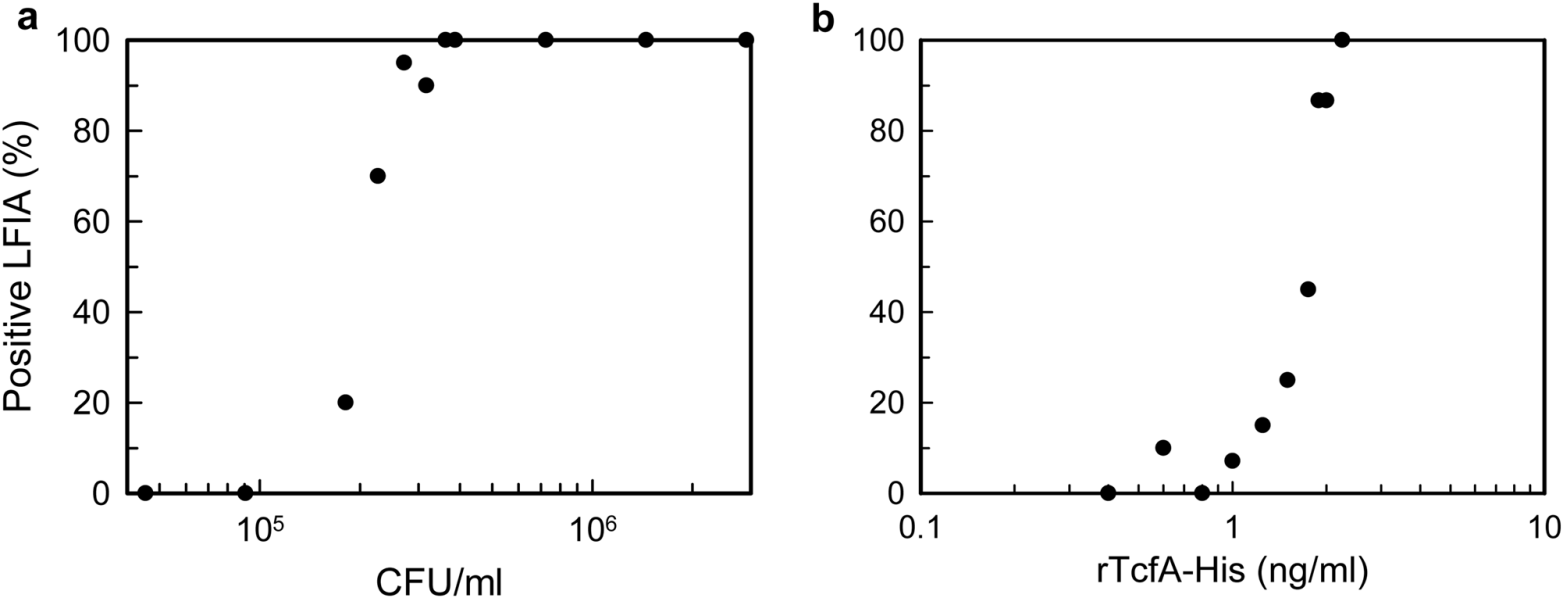
Analytical sensitivity of the pertussis LFIA with viable *B. pertussis* cells in PBS and with rTcfA-His. (**A**) Viable *B. pertussis* cells were suspended in PBS at 11 different concentrations, and each concentration was tested with 20 LFIAs (n = 220). (**B**) rTcfA-His was diluted in PBS at 10 different concentrations, and each concentration was tested with at least 10 LFIAs (n = 144). Summary statistics representing the percentage of LFIA replicates visually interpreted as positive by three of three blinded readers at each concentration are displayed.

The analytical sensitivity of the LFIA was determined with viable *B. pertussis* cells (Tohama I) in PBS and with rTcfA-His (Fig. 4). For each sample type, at least 10 concentrations were evaluated with at least 10 LFIA replicates per concentration. Nonlinear regression analysis using a 4-parameter logistics model (SAS statistical package, version 9.4) on the individual LFIA replicates (n = 220 for *B. pertussis* cells and n = 144 for rTcfA-His) was used to calculate the lowest analyte concentration at which 95% of the LFIA replicates run would be interpreted as positive by three of three blinded readers (i.e. the assay’s limit of detection, LOD). The LOD for the LFIA with viable *B. pertussis* cells (Tohama I) was 3.0 × 10^5^ CFU/mL (95% confidence interval [CI], 2.8 × 10^5^ to 3.4 × 10^5^ CFU/mL) (Fig. 4A and Supplementary Fig. S5). The LOD with rTcfA-His was 2.3 ng/mL (95% CI, 2.2 to 2.6 ng/mL) (Fig. 4B and Supplementary Fig. S5).

**Figure 5:**
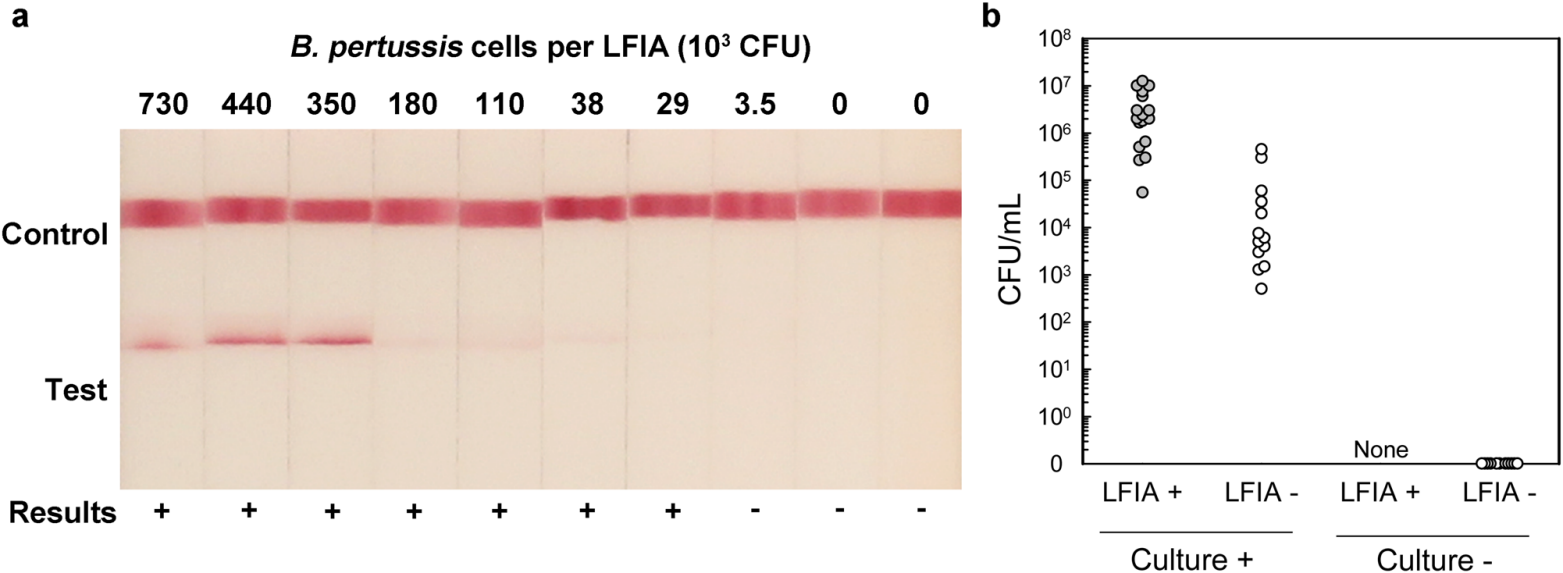
Reactivity of pertussis LFIA with NP washes from baboons directly challenged with *B. pertussis*. (**A**) Representative LFIA images from NP specimens having a range of bacterial burdens are shown along with the visual interpretation of results by three blinded readers. (**B**) LFIA results (single replicate) are indicated. Of the 11 culture negative (0 CFU/mL) NP wash specimens, all were negative on the LFIA.

LFIA performance was assessed with NP wash specimens from baboons directly challenged with *B. pertussis* (D420) via a well-established non-human primate model^1,41^. Building on the inclusivity testing of Supplementary Fig. S3, D420 was the ninth strain of eleven tested strains that reacted with the LFIA. A photograph of representative LFIAs run with NP washes having a range of bacterial burdens as well as the visual interpretation of those LFIAs by three blinded readers are shown (Fig. 5A). Each NP wash (n = 41) was tested on duplicate LFIAs, with the exception of 4 specimens for which low sample volume permitted only a single replicate. The LFIA produced no false-positives with any of the 11 NP washes containing 0 CFU/mL (100% specificity) (Fig. 5B). All NP washes with ≥ 4.9 × 10^5^ CFU/mL were positive, and all NP washes with ≤ 3.4 × 10^4^ CFU/mL were negative (Fig. 5B). The lowest concentration scored as positive was 5.4 × 10^4^ CFU/mL (3.2 × 10^3^ CFU/test) (Fig. 5B). The LOD with baboon NP washes (n = 78 tests) was 6.2 × 10^5^ CFU/mL (95% CI, 4.9 × 10^5^ to 1.1 × 10^6^ CFU/mL) (Fig. 5 and Supplementary Fig. S6).

### LFIA assessment with patient NP swab specimens

NP swab specimens from nine human patients with suspected pertussis were evaluated by LFIA with three blinded readers (Table 2, Supplementary Fig. S7). Four specimens that were negative by qPCR were also negative by LFIA (Table 2). Similarly, two qPCR-positive specimens with Ct values corresponding to ≥ 1.8 × 10^6^ genomes/mL were also positive by LFIA (Table 2). Two specimens from qPCR-positive patients had Ct values equivalent to 3.6 × 10^5^ and 8.1 × 10^4^ genomes/mL, resulting in split visual interpretations of the LFIA by the blinded readers: two of three readers interpreted the specimen with 3.6 × 10^5^ genomes/mL as positive, and one of three readers interpreted the specimen with 8.1 × 10^4^ genomes/mL as positive (Table 2). The concentration range covered by these values is within the concentration range that also produced both positive and negative LFIAs in the baboon NP wash testing (i.e. 3.4 × 10^4^ to 4.9 × 10^5^ CFU/mL, Fig. 5).

**Table 2:** Nasopharyngeal swab specimens from nine patients with suspected pertussis were evaluated by qPCR and LFIA.

Patient	qPCR Ct	Calculated^a^ genomes/mL	qPCR diagnosis^b^	LFIA result^c^
1	7.4	1.1 × 10^7^	Positive	Positive
2	10.2	1.8 × 10^6^	Positive	Positive
3	12.7	3.6 × 10^5^	Positive	Indeterminant^d^
4	15	8.1 × 10^4^	Positive	Indeterminant^e^
5	18.9	6.5 × 10^3^	Positive	Negative
6	ND^f^	NA^g^	Negative	Negative
7	ND	NA	Negative	Negative
8	ND	NA	Negative	Negative
9	ND	NA	Negative	Negative

^a^Observed qPCR Ct values for sample loaded onto LFIA were converted to genomes/mL based on a standard curve with purified *B. pertussis* genomic DNA.

^b^Ct values < 35 were diagnosed as positive; qPCR was performed as described^52^.

^c^Consensus visual interpretation by three blinded readers. Where all three readers were not in agreement, independent reader scores are listed (d and c).

^d^Two readers scored the LFIA as positive; one reader scored the LFIA as negative.

^e^One reader scored the LFIA positive; two readers scored the LFIA as negative.

^f^No Ct value because target was not detected.

^g^Not applicable because there was no Ct value.

## Discussion

There is an unmet clinical need for rapid, point-of-care diagnosis of pertussis. This study identified TcfA as a biomarker that could be used to detect *B. pertussis* in < 20 min by LFIA. Because *B. pertussis* cells are present in NP specimens, we pursued antibodies that recognized formaldehyde-inactivated *B. pertussis* cells (Table 1). Moreover, TcfA has a secreted and a cell surface associated isoform^24^. Thus, to increase the potential clinical sensitivity of the LFIA, we targeted regions conserved in both isoforms (i.e. amino acids 40–374)^24^ for antibody development. All 28 TcfA MAbs bound formaldehyde-inactivated *B. pertussis* cells, and all TcfA MAbs bound epitopes conserved in both TcfA isoforms (Fig. 1 and Supplementary Fig. S1 and S2).

LFIA testing demonstrated that some MAbs formed functional LFIA pairs only as test line MAbs (*e.g.* MAbs 15A9, 14D9, and 19F9) or only as gold conjugate MAbs (*e.g.* MAbs 7A10 and 22B7) (Fig. 2). Some MAbs worked equally well in both positions (*e.g.* MAbs 13E11 and 18B2) (Fig. 2). The least amount of background and strongest signal was observed with the LFIA containing MAb 10B1 as gold conjugate and a combination of MAbs 13E11 and 14D12 at the test line (Figs. 2 and 3).

This LFIA configuration was highly specific for *B. pertussis* when evaluated with 40 other microorganisms potentially found in the nasopharynx, including *B. parapertussis, B. holmesii*, and *B. bronchiseptica* (Supplementary Table S1). Infections with these *Bordetella* species are rarer than infections with *B. pertussis*, and optimal treatment regimens for them have yet to be established^9, 42,43^. Where clinical guidelines do exist, they generally call for different actions to be triggered for a diagnosis of *B. pertussis* than for other *Bordetella* species. Thus, the lack of cross-reactivity of the pertussis LFIA with *B. bronchiseptica, B. parapertussis,* and *B. holmesii* (Supplementary Table S1) may be advantageous for guiding appropriate actions regarding patient treatment, post-exposure prophylaxis, and public health notifications.

Inclusivity testing indicated that the LFIA was reactive with multiple strains of *B. pertussis*. Specifically, the LFIA reacted with 9 of 11 *B. pertussis* strains and two untyped isolates from patient NP specimens (Supplementary Fig. S3, Fig. 5, and Table 2). One of the two strains that did not react with the LFIA was 18323, which was isolated over 70 years ago^32^. The 25 additional amino acids in the *tcfA1* allele of 18323^34^ (relative to the *tcfA2* allele found in Tohama I) may change the local secondary structure of TcfA near the epitope of MAb 10B1 (Supplementary Fig. S4), which may explain the LFIA’s lack of reactivity with this strain. The other strain that did not react with the LFIA was 5374(3747), which was isolated over 60 years ago^33^. To our knowledge, *tcfA* sequence data for 5374(3747) has not been published. Notably, this strain is known to differ from more recent clinical isolates due to its lack of expression of the type III secretion system (TTSS) effector, Bsp22^44^. Like TcfA, the TTSS is a virulence factor that may be important for efficient respiratory tract colonization^24,44^.

The LFIA’s inclusivity was further analyzed in silico with sequences from 954 *B. pertussis* isolates from 12 countries^34–40^. In sum, of 954 *B. pertussis* isolates (including 10 *tcfA* deletion and frameshift mutants identified through a dedicated search for isolates with deficient TcfA expression^39^), we would predict that the pertussis LFIA would be highly inclusive and would react with 98% of them (i.e. 935 of 954 isolates contained *tcfA2, tcfA3,* or *tcfA4*).

Both high inclusivity and high analytical sensitivity are necessary for high clinical sensitivity. Two reports describing the bacterial burden in NP washes from four infants, all aged less than 3 months, have been published^16,25^. These specimens contained 8 × 10^10^ genomes/mL, 1.2 × 10^9^ genomes/mL, 1 × 10^8^ CFU/mL, and 7.5 × 10^7^ CFU/mL^16,25^. These bacterial burdens are 100-fold to 125,000-fold greater than the LOD of the pertussis LFIA with baboon NP washes, and 250-fold to 250,000-fold greater than the LFIA’s LOD with *B. pertussis* cells in PBS, which further suggests that using TcfA as a biomarker for LFIA-based detection of infant pertussis may be feasible.

Specimen type will likely be an important variable in assay performance. Although both NP swabs and NP washes are compatible specimen types for the pertussis LFIA (Table 2 and Fig. 5), NP washes would likely be the preferred sample specimen for clinical use due to the higher concentration of bacteria reported in infant NP washes *vs.* NP swab extracts^16,25,45–47^. In a direct comparison of specimen collection methods, washes were found to be a much more sensitive sampling method than swabs for PCR-mediated detection of *B. pertussis*: in cases where a patient had both a nasal wash and a pernasal swab collected at the same time and the wash was positive, the corresponding swab was positive only approximately 50% of the time^48^.

Patient age may also impact assay performance. Younger patients have generally been found to have higher bacterial burdens at the time of specimen collection than older patients^45,46^. This may limit the pertussis LFIA’s clinical utility to younger patients (*e.g.* infants). However, an infant-use-only indication might not materially reduce the public health impact of the assay, since (i) infants are at greater risk of hospitalization, morbidity, and mortality from pertussis than older patients, and (ii) infants require more healthcare visits on average to receive a pertussis diagnosis than do older patients^14,15^.

Our examination of human samples provided strong proof-of-concept that TcfA is a biomarker for infection, but likely underestimated the potential sensitivity of the LFIA for detection of infection. First, the remnant patient specimens we analyzed were from swabs, not washes. Second, the swabs had been placed into 1 mL of Amies medium instead of a minimal volume (*e.g.* 0.1 mL). Third, a further two-fold dilution of the specimens into SDS/PBS extraction buffer was required to mitigate interference of the Amies medium with the LFIA. In the future, a prospective clinical trial with NP washes from symptomatic patients will be needed to rigorously determine the assay’s clinical sensitivity and specificity as a function of patient age and disease stage.

In conclusion, we have identified a novel biomarker (TcfA) for detecting *B. pertussis* infection by LFIA. The LFIA’s LOD with NP washes from a non-human primate model and reactivity with NP swab specimens from human patients raises the possibility that a proven diagnostic approach for many other infectious diseases (i.e. LFIA) can be brought to bear on pertussis. A pertussis LFIA would differ from existing pertussis tests in that it would (i) require no electricity or specialized equipment, (ii) provide results in less than 20 min, and (iii) eliminate the need to send specimens to the laboratory. A rapid test that could be used at the point-of-care even in resource limited settings would enable immediate patient triage and prompt initiation of appropriate antibiotic therapy, which in turn should help reduce patient morbidity, limit further transmission, and save infant lives.

## Methods

### *B. pertussis* proteins

Amino acids 40–374 of *B. pertussis* TcfA (Tohama I, UniprotKB Q79GX8) were cloned into pQE-30-Xa (Qiagen), which contains an N-terminal 6XHis-tag followed by a Factor Xa cleavage site. rTcfA-His was purified over HisPur Cobalt columns (Pierce). To generate tag-free rTcfA, purified rTcfA-His was treated with Factor Xa (New England Biolabs), then 1,5-Dansyl-Glu-Gly-Arg-chloromethyl (EMD Millipore), and passed again over HisPur Cobalt columns. Endogenous *B. pertussis* adenylate cyclase antigen and filamentous hemagglutinin were purchased from List Biological Laboratories (Campbell, CA).

### *B. pertussis* strains

*B. pertussis* strains Tohama I, 18323, H792, H735, 5374[3747], and CNCTC Hp 12/63[623] were obtained from ATCC (Manassas, VA); A639 and E431 from Zeptometrix (Buffalo, NY); 165 from List Biological Laboratories; and H973 from BEI Resources, NIAID, NIH (Manassas, VA).

### Culture and formaldehyde-inactivation of *B. pertussis*

With the exception of 165, all *B. pertussis* strains were cultured at 37ºC on Bordet-Gengou plates with defibrinated sheep blood^49^. Bacteria were aseptically washed from the plates with sterile phosphate buffered saline (PBS) and collected by centrifugation. Unfixed aliquots were prepared by bringing the cell suspension to an OD_600_ of 1 with PBS pH 7.4 and storing at -80ºC. For formaldehyde-fixed aliquots, cells were brought to an OD_600_ of 60–70 with PBS pH 7.8 and formalin was added to a final concentration of 0.14% before incubating at 37 °C for 5 days^50^. Cells were then washed in PBS, pH 7.4, brought to an OD_600_ of 10–25, and stored at − 80 °C.

Strain 165 was cultured by List Biological Laboratories in modified Stainer-Scholte liquid medium. After centrifugation, the supernatant was 0.2 µm-filtered and stored at -80ºC, while the bacterial pellet was resuspended in PBS, fixed with formalin, and washed with PBS as described above.

### Antibody production

Immunization of rabbits for polyclonal antibody (pAb) production and of mice for MAb production was approved by the University of Nevada, Reno Institutional Animal Care and Use Committee (IACUC); all experiments were performed in accordance with relevant guidelines and regulations. ProSci, Inc. (Poway, CA) prepared pAbs by immunizing rabbits with peptide immunogens coupled to keyhole limpet hemocyanin (KLH). The resulting pAbs were affinity-purified over peptide-resin columns.

MAbs were prepared in-house by immunizing mice with (i) TcfA peptides conjugated to KLH, (ii) formaldehyde-inactivated *B. pertussis* cells (Tohama I) in PBS, or (iii) rTcfA, peptide:KLH conjugates, and formaldehyde-inactivated *B. pertussis* cells. Splenocytes were isolated and cryopreserved as described^51^. Hybridomas were generated from cryopreserved splenocytes via standard protocols and subjected to multiple rounds of cloning by limiting dilution. MAbs were purified from cell culture supernatant using rProtein A (GE).

### ELISA

Bovine serum albumin (BSA) and TcfA peptides conjugated to BSA were diluted in 50 mM carbonate, pH 9.6. All other antigens were diluted in PBS, pH 7.4. For ELISAs with MAbs, microtiter plates were incubated with antigens overnight, washed with PBS-Tween (PBS containing 0.05% Tween-20), and blocked for 90 min with blocking buffer (PBS containing 0.5% Tween-20 and 5% w/v non-fat powdered milk) at room temperature (RT). Plates were washed, incubated for 90 min at RT with antibodies in blocking buffer, washed again, then incubated 90 min at RT with horseradish peroxidase-labeled (HRP-labeled) goat anti-mouse secondary antibodies (Southern Biotech, Birmingham, AL and Jackson ImmunoResearch Laboratories, Inc., West Grove, PA) in blocking buffer. After a final wash, plates were incubated for 30 min at RT with tetramethylbenzidine substrate (SeraCare, Milford, MA). The reaction was stopped with 1 M H_3_PO_4_, and plates were read at OD_450_.

Indirect and direct ELISAs with rabbit pAbs were performed similarly except for the following changes: blocking buffer was PBS containing 0.5% Tween-20 and 1% (v/v) normal rabbit serum (MP Biomedicals), incubations were done for 1 h at 37ºC, washes after the blocking step and after the primary antibody incubation were done with blocking buffer, and rat anti-rabbit IgG-HRP (Southern Biotech) was used. Secondary antibody steps were omitted when using HRP-conjugated rabbit pAbs.

For antigen-capture ELISAs with rabbit pAbs, plates were coated overnight with affinity purified pAbs, washed with PBS-Tween, blocked with 0.5% Tween-20 and 1% (v/v) normal rabbit serum for 90 min at RT, then incubated with antigen in blocking buffer for 90 min at RT. After washing in PBS-Tween, plates were incubated with substrate, stopped, and read as above. Data was analyzed with SoftMax Pro (version 6.4), using a log–log plot.

### MAb epitope mapping

A tiled peptide library covering amino acids 40-374 of TcfA was constructed (Mimotopes, Melbourne, Australia). The library contained 108 peptides (15mers, offset by three amino acids). Peptides were biotinylated with an “SGSG” spacer at the N-terminus and an amide at the C-terminus. Standard binding streptavidin-coated microtiter plates pre-blocked with bovine serum albumin (BSA) (ThermoScientific Pierce) were incubated for 1 h with shaking with peptides at 0.1 µg/mL in PBS containing 0.1% (w/v) BSA. Plates were washed with PBS containing 0.05% Tween-20 (PBS-Tween), incubated for 1 h with shaking with purified MAbs at 1 µg/mL in PBS containing 2% (w/v) BSA, washed again, and incubated for 1 h with shaking with goat anti-mouse IgG-Fc-HRP (Jackson ImmunoResearch Laboratories, Inc., West Grove, PA) in PBS containing 2% (w/v) BSA. Plates were washed with PBS-Tween, then PBS, then incubated with tetramethylbenzidine substrate (SeraCare, Milford, MA) for 6 min before stopping with 1 M H_3_PO_4_ and reading at OD_450_.

### LFIA construction

For identification of compatible MAb pairings, LFIAs were prepared for wet testing by covalently conjugating MAbs to 40 nm InnovaCoat Gold (Expedeon, Cambridge, UK), and loading 5µL of gold conjugate at an OD_530_ of 10 onto the conjugate pad (Fusion 5, GE), which was blocked with 0.01 M borate buffer, pH 8, containing 0.25% Triton-X-100. The sample pad (Standard 14, GE) was blocked identically. The test line was sprayed onto FF120HP nitrocellulose (GE) with the relevant MAb at 1 mg/mL in PBS, and the control line was sprayed with goat anti-mouse Ig, Human adsorbed (Southern Biotech) at 1 mg/mL in PBS using a BioDot XYZ3050 Biojet system at 1 µL/cm. LFIAs were assembled onto 60 mm tall adhesive backing cards (DCN Diagnostics, Carlsbad, CA) with a CFSP203000 (Millipore) wicking pad and cut into 4-mm-wide strips.

All other testing was done with LFIAs optimized to the following specifications. MAb 10B1 was passively adsorbed onto 40 nm colloidal gold particles (DCN Diagnostics) and brought to an OD_530_ of 20 in PBS, pH 8 containing 0.5% (v/v) Pluronic F127, 1% (w/v) BSA, 20% (w/v) sucrose, and 5% (w/v) trehalose. Gold conjugated MAb was dispensed using a BioDot XYZ3050 Airjet system at 10µL/cm onto a Fusion 5 conjugate pad (blocked as above) and then dried at 37ºC. Antibody solutions for the test line (1 mg/mL MAb 13E11 and 1 mg/mL MAb 14D12) and control line were contact-dispensed onto FF80HP nitrocellulose (GE) using a BioDot XYZ3050 Biojet system at 1µL/cm. LFIAs were assembled and cut as above with CFSP203000 wicking pads and blocked Standard 14 sample pads.

### LFIA testing

For identification of MAb pairs, samples in PBS or sodium dodecyl sulfate (SDS) in PBS were loaded onto LFIA sample pads, and LFIAs were run in microtiter plates containing 200µL PBS as chase buffer for 20 min. LFIAs were read visually and signal was quantified with a Qiagen ESEQuant Lateral Flow Reader with Lateral Flow Studio Software suite (version 3.3.8). For all other testing, samples were prepared in a final concentration of 0.25% (w/v) SDS in PBS and incubated at room temperature for 5 min. Then 60µL was loaded onto each LFIA sample pad, and LFIAs were run in 5 mL open-cap tubes containing 200µL PBS for 15 min. LFIAs were read as above.

### LFIA evaluation with nasopharyngeal (NP) specimens

Remnant NP washes from baboons directly challenged with *B. pertussis* (D420 strain) and CFU/mL data were provided by Dr. Tod Merkel at the Food and Drug Administration’s Center for Biological Evaluation and Research. Baboon challenge experiments were approved by the FDA’s IACUC and were performed in accordance with relevant guidelines and regulations. Remnant, deidentified NP swab specimens in Eswab Amies buffer (BD, Franklin Lakes, NJ) from patients with suspected pertussis infection and qPCR results^52^ were provided by Dr. James Dunn at Texas Children’s Hospital. NP washes were combined with 10% SDS, while NP swab specimens were combined with 0.5% SDS for a final concentration of 0.25% SDS for LFIA analysis.

## Supplementary information

Supplementary Information.

## Data Availability

All data generated or analyzed during this study are included in this published article (and its Supplementary Information files).
