# Supplementary material for: Tracheal colonization factor A (TcfA) is a biomarker for rapid and specific detection of Bordetella pertussis
Source: Sci Rep. 2020 Sep 14;10:15002. doi: 10.1038/s41598-020-72092-6 (PMC7490692; doi:10.1038/s41598-020-72092-6)
Supplement: Supplementary file 1 — Supplementary Information. [file 41598_2020_72092_MOESM1_ESM.pdf]

Supplementary Material

Tracheal colonization factor A (TcfA) is a biomarker for rapid and specific detection of *Bordetella pertussis*

Amanda R. Burnham-Marusich,[a,#] Ryan K. Olsen,[a] Jacqueline Scarbrough,[a,b] Alexander Kvam,[b] Wei Yang,[c] Lindsey Zimmerman,[d] James J. Dunn,[e] Tod Merkel,[d] and Thomas R. Kozel[a,b]

[a]DxDiscovery; Reno, Nevada, USA

[b]Department of Microbiology & Immunology, University of Nevada, Reno School of Medicine; Reno, Nevada, USA

[c]Department of Community Health Sciences, University of Nevada, Reno; Reno, Nevada, USA

[d]Center for Biological Evaluation and Research, Food and Drug Administration; Silver Spring, Maryland, USA

[e]Texas Children’s Hospital, Houston; TX, USA

Supplementary Table S1. The LFIA showed no cross-reactivity with the listed microorganisms.

Supplementary Fig. S1. Anti-TcfA MAb reactivity by ELISA with *B. pertussis* antigens in the solid phase.

Supplementary Fig. S2. Diagram of MAb minimal linear epitopes on TcfA.

Supplementary Fig. S3. Reactivity of pertussis LFIA with multiple strains of *B. pertussis*.

Supplementary Fig. S4. Pairwise alignment of the *tcfA1* and *tcfA2* alleles with LFIA MAb epitopes.

Supplementary Fig. S5. Non-linear regression analysis of the analytical sensitivity of the LFIA with viable *B. pertussis* cells in PBS and with rTcfA-His.

Supplementary Fig. S6. Non-linear regression analysis of baboon NP wash specimens with the LFIA.

Supplementary Fig. S7. Reactivity of pertussis LFIA with patient NP swab specimens.

Supplementary Table S1. The LFIA showed no cross-reactivity with the listed microorganisms[a]

Acinetobacter baumanni	*Enterococcus faecium (Z265)*
Acinetobacter calcoaceticus	*Escherichia coli (O157)*
Acinetocacter lwoffii	*Haemophilus influenzae*
Aspergillus fumigatus	*Haemophilus parainfluenzae (NCTC 7857)*
Bacillus cereus	*Legionella pneumophila (Philadelphia)*
Bacillus subtilis	*Moraxella catarrhalis*
Bacteroides fragilis	*Morganella morganii (Z098)*
Bordetella bronchiseptica (NCTC 452)	*Mycoplasma pneumonia (M129)*
Bordetella bronchiseptica (RB50)	*Proteus mirabilis (Z050)*
Bordetella holmesii (F061)	*Proteus vulgaris (Z129)*
Bordetella parapertussis (A747)	*Pseudomonas aeruginosa*
Bordetella parapertussis (C510)	*Staphylococcus epidermidis*
Candida albicans	*Stenotrophomonas maltophilia (Z074)*
Candida glabrata	*Streptococcus agalactiae (Z019)*
Citrobacter amalonaticus	*Streptococcus dysgalactiae (Z068)*[b]
Citrobacter freundii	*Streptococcus mitis*
Citrobacter koseri	*Streptococcus mutans (Z072)*
Cornebacterium diphtheria	*Streptococcus pneumoniae*
Enterobacter aerogenes	*Streptococcus pyogenes*
Enterobacter cloacae	*Streptococcus salivarius (Z127)*
Enterococcus faecalis (Z346)	*Streptococcus sanguinis (Z089)*

[a]Testing was done with triplicate LFIAs using 3.3 x 10^7 CFU/mL

[b]Initial testing was positive. Subsequent testing with i) the same lot of *S. dysgalactiae* subsp. *equisimilis*, ii) a different lot of *S. dysgalactiae* subsp. *equisimilis,* iii) a different strain of *S. dysgalactiae* subsp. *equisimilis* (ATCC 35666), and iv) *S. dysgalactiae* subsp. *dysgalactiae,* all across two different LFIA lots, was negative.

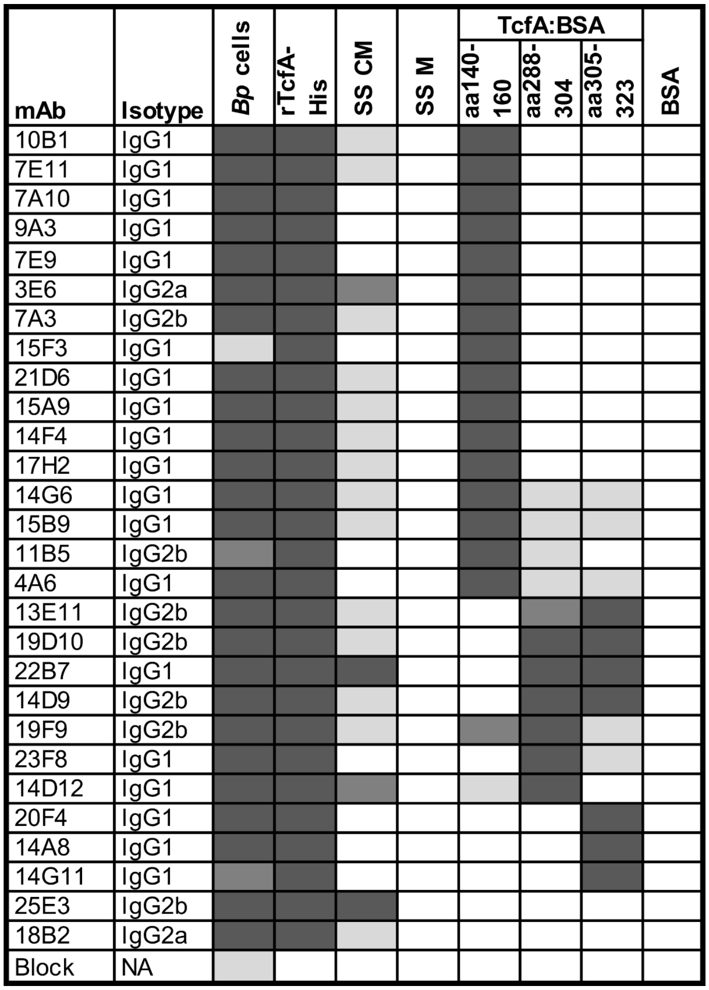

Supplementary Fig. S1. Anti-TcfA MAb reactivity by ELISA with *B. pertussis* antigens in the solid phase. Heat map shading corresponds to signal intensity (absorbance at 450nm). White indicates signal intensity ≤ 0.2, light gray from > 0.2 to 1, medium gray from >1 to 2, and dark gray from >2 to 4. Data is the average of three independent experiments. *B. pertussis* (*Bp*) cells (Tohama I) were formaldehyde-inactivated and used at an OD_{600} of 1.0, rTcfA-His was used at 1µg/mL, BSA conjugates of the indicated TcfA peptides and BSA were used at 0.1µg/mL. SS CM = 1:128 dilution in PBS of 0.2µm-filtered supernatant from *B. pertussis* (Tohama I) Stainer Scholte liquid cultures. SS M = 1:128 dilution in PBS of Stainer Scholte uninocculated medium.

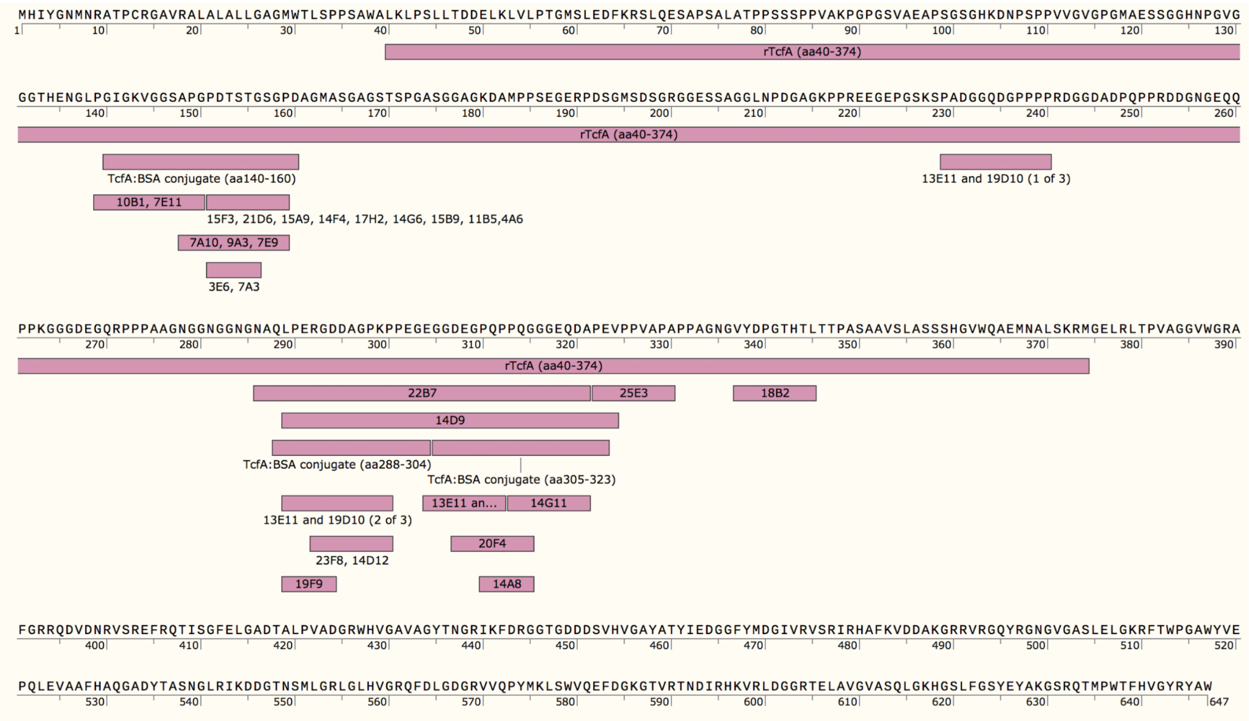

Supplementary Fig. S2. MAb minimal linear epitopes on TcfA. Minimal linear epitopes were determined by indirect ELISA with a tiled library of biotinylated TcfA peptides. Labeled features below the amino acid sequence indicate: i) the region of TcfA encompassed by the expressed, recombinant TcfA protein, ii) the three TcfA protein fragments used for initial epitope binning of MAbs, and iii) the minimal linear epitope sequences for each MAb. Minimal linear epitope sequences were defined as the minimal overlapping peptide sequence for wells with an OD_{450} greater than 0.75 (and in a series of two or more such adjacent wells). MAbs 22B7 and 14D9 reacted with a long series of peptides such that the first peptide in the series did not overlap with the last peptide. For these MAbs, the peptide sequence defined by the entire series of reactive wells is listed.

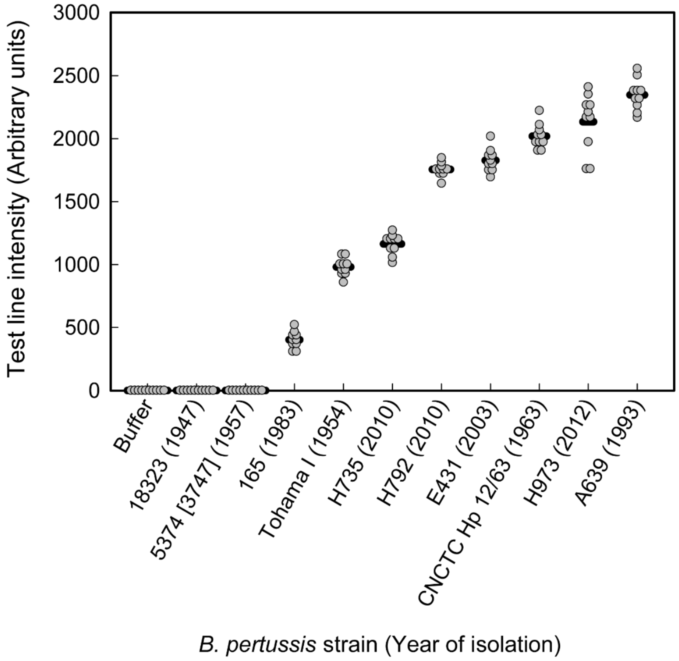

Supplementary Fig. S3. Reactivity of pertussis LFIA with multiple strains of *B. pertussis*. Formaldehyde-inactivated *B. pertussis* cells of the listed strains were incubated for 5 min with 0.25% SDS in PBS, pH 7.4 at an OD_{600} of 0.1 (equivalent to 4.8 x 10^7 CFU/mL). Results are reported as test line intensity. For each strain, the mean of the 10 LFIA replicates tested is indicated with a black horizontal line.

```
               10        20        30        40        50        60
tcfA1  MHIYGNMNRATPCRGAVRALALALLGAGMWTLSPPSAWALKLPSLLTDDELELVLPTGMS
       ::::::::::::::::::::::::::::::::::::::::::::::::::::.::::::::
tcfA2  MHIYGNMNRATPCRGAVRALALALLGAGMWTLSPPSAWALKLPSLLTDDELKLVLPTGMS
               10        20        30        40        50        60

               70        80        90       100       110       120
tcfA1  LEDFKRSLQESAPSALATPPSSSPPVAKPGPGSVAEAPSGSGHKDNPSPPVVGVGPGMAE
       ::::::::::::::::::::::::::::::::::::::::::::::::::::::::::::
tcfA2  LEDFKRSLQESAPSALATPPSSSPPVAKPGPGSVAEAPSGSGHKDNPSPPVVGVGPGMAE
               70        80        90       100       110       120

              130       140       150       160       170       180
tcfA1  SSGGHNPGVGGGTHENGLPGIGKVGGSAPGPGGLGRNDENSESSLNPGTLGPSPGPDTST
       :::::::::::::::::::::::::::::::                         ::::
tcfA2  SSGGHNPGVGGGTHENGLPGIGKVGGSAPGP-------------------------DTST
              130       140       150

              190       200       210       220       230       240
tcfA1  GSGPDAGMASGAGSTSPGASGGAGKDAMPPSEGERPDSGMSDSGRGGESSAGGLNPDGAG
       ::::::::::::::::::::::::::::::::::::::::::::::::::::::::::::
tcfA2  GSGPDAGMASGAGSTSPGASGGAGKDAMPPSEGERPDSGMSDSGRGGESSAGGLNPDGAG
         160       170       180       190       200       210

              250       260       270       280       290       300
tcfA1  KPPREEGEPGSKSPADGGQDGPPPPRDGGDADPQPPRDDGNGEQQPPKGGGDEGQRPPPA
       ::::::::::::::::::::::::::::::::::::::::::::::::::::::::::::
tcfA2  KPPREEGEPGSKSPADGGQDGPPPPRDGGDADPQPPRDDGNGEQQPPKGGGDEGQRPPPA
         220       230       240       250       260       270

              310       320       330       340       350       360
tcfA1  AGNGGNGGNGNAQLPERGDDAGPKPPEGEGGDEGPQPPQGGGEQDAPEVPPVAPAPPAGN
       ::::::::::::::::::::::::::::::::::::::::::::::::::::::::::::
tcfA2  AGNGGNGGNGNAQLPERGDDAGPKPPEGEGGDEGPQPPQGGGEQDAPEVPPVAPAPPAGN
         280       290       300       310       320       330

              370       380       390       400       410       420
tcfA1  GVYDPGTHTLTTPASAAVSLASSSHGVWQAEMNALSKRMGELRLTPVAGGVWGRAFGRRQ
       ::::::::::::::::::::::::::::::::::::::::::::::::::::::::::::
tcfA2  GVYDPGTHTLTTPASAAVSLASSSHGVWQAEMNALSKRMGELRLTPVAGGVWGRAFGRRQ
         340       350       360       370       380       390

              430       440       450       460       470       480
tcfA1  DVDNRVSREFRQTISGFELGADTALPVADGRWHVGAVAGYTNGRIKFDRGGTGDDDSVHV
       ::::::::::::::::::::::::::::::::::::::::::::::::::::::::::::
tcfA2  DVDNRVSREFRQTISGFELGADTALPVADGRWHVGAVAGYTNGRIKFDRGGTGDDDSVHV
         400       410       420       430       440       450

              490       500       510       520       530       540
tcfA1  GAYATYIEDGGFYMDGIVRVSRIRHAFKVDDAKGRRVRGQYRGNGVGASLELGKRFTWPG
       ::::::::::::::::::::::::::::::::::::::::::::::::::::::::::::
tcfA2  GAYATYIEDGGFYMDGIVRVSRIRHAFKVDDAKGRRVRGQYRGNGVGASLELGKRFTWPG
         460       470       480       490       500       510

              550       560       570       580       590       600
tcfA1  AWYVEPQLEVAAFHAQGADYTASNGLRIKDDGTNSMLGRLGLHVGRQFDLGDGRVVQPYM
       ::::::::::::::::::::::::::::::::::::::::::::::::::::::::::::
tcfA2  AWYVEPQLEVAAFHAQGADYTASNGLRIKDDGTNSMLGRLGLHVGRQFDLGDGRVVQPYM
         520       530       540       550       560       570

              610       620       630       640       650       660
tcfA1  KLSWVQEFDGKGTVRTNDIRHKVRLDGGRTELAVGVASQLGKHGSLFGSYEYAKGSRQTM
       ::::::::::::::::::::::::::::::::::::::::::::::::::::::::::::
tcfA2  KLSWVQEFDGKGTVRTNDIRHKVRLDGGRTELAVGVASQLGKHGSLFGSYEYAKGSRQTM
         580       590       600       610       620       630

              670
tcfA1  PWTFHVGYRYAW
       ::::::::::::
tcfA2  PWTFHVGYRYAW
         640
```

Supplementary Fig. S4. Pairwise alignment of the *tcfA1* and *tcfA2* alleles with pertussis LFIA MAb epitopes. The *tcfA1* and *tcfA2* alleles (GenBank accession numbers AAC43453.1 and CAA08832.2, respectively) were aligned using LALIGN[1]. The minimal linear epitopes of MAb 10B1 (the pertussis LFIA's gold conjugate detector MAb) is indicated with blue highlighting. The minimal linear epitopes of MAbs 14D12 and 13E11 (the pertussis LFIA's test line capture MAbs) are indicated with yellow highlighting.

[1] Madeira, F. *et al*. The EMBL-EBI search and sequence analysis tools APIs in 2019. *Nucleic Acids Res* **47**, W636-W641, doi:10.1093/nar/gkz268 (2019).

a

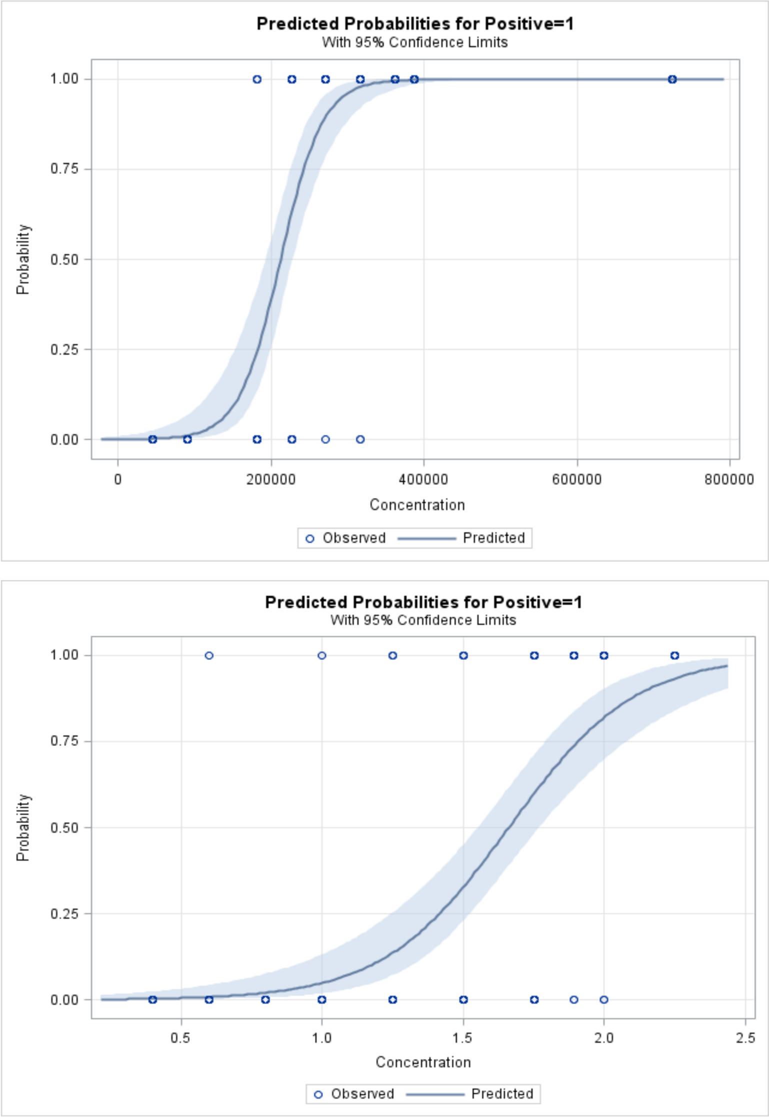

b

Supplementary Fig. S5. Non-linear regression analysis of the analytical sensitivity of the pertussis LFIA with viable *B. pertussis* cells in PBS and with rTcfA-His. A) Viable *B. pertussis* cells were suspended in PBS at 11 different concentrations (CFU/mL), and each concentration was tested with 20 LFIAs (n=220). B) rTcfA-His was diluted in PBS at 10 different concentrations (ng/mL), and each concentration was tested with at least 10 LFIAs (n=144). The concentration (CFU/mL or ng/mL) of the sample was plotted vs. the outcome of the LFIA (a positive LFIA result was recorded as

“1” whereas a negative LFIA result was “0”). Nonlinear regression analysis using a 4-parameter logistics model was used to determine the line of best fit and to calculate the lowest analyte concentration at which 95% of the LFIA replicates run would be interpreted as positive by three of three blinded readers (*i.e.* the assay’s limit of detection, LOD). The LOD for the pertussis LFIA with viable *B. pertussis* cells was 2.97 x 10^5 CFU/mL (95% confidence interval [CI], 2.8 x 10^5 to 3.4 x 10^5 CFU/mL). The LOD with rTcfA-His was 2.3 ng/mL (95% CI, 2.2 to 2.6 ng/mL).

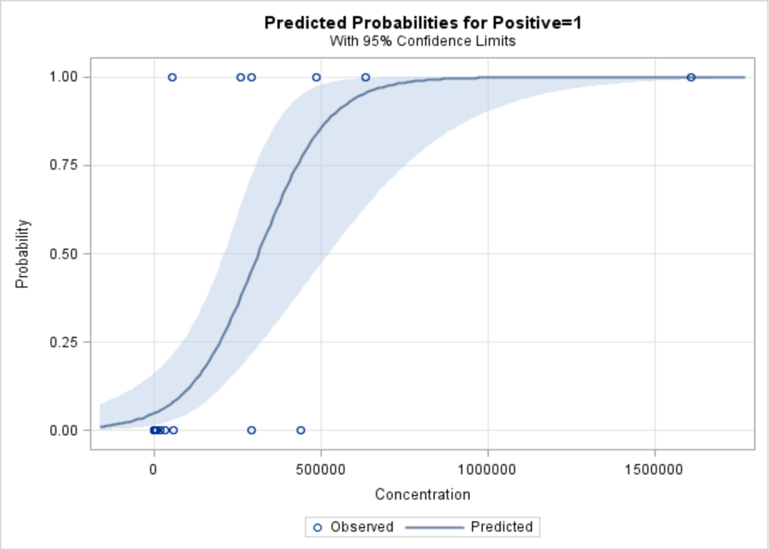

Supplementary Fig. S6. Non-linear regression analysis of baboon NP wash specimens with the pertussis LFIA. Remnant NP wash specimens from baboons directly challenged with *B. pertussis* (strain D420) were incubated with 0.25% SDS for 5 min at room temperature and then developed on the LFIA for 15 min. Each NP wash specimen (n=41) was tested on duplicate LFIAs, with the exception of 4 specimens for which low sample volume permitted only a single LFIA replicate. The concentration (CFU/mL) of the NP specimen was plotted vs. the outcome of the LFIA (a positive LFIA result was recorded as “1” whereas a negative LFIA result was “0”). Nonlinear regression analysis using a 4-parameter logistics model was used to determine the line of best fit and to calculate the LFIA’s LOD. The LOD for the pertussis LFIA with the baboon NP wash specimens was 6.2×10^5 CFU/mL (95% CI, 4.9×10^5 to 1.1×10^6 CFU/mL).

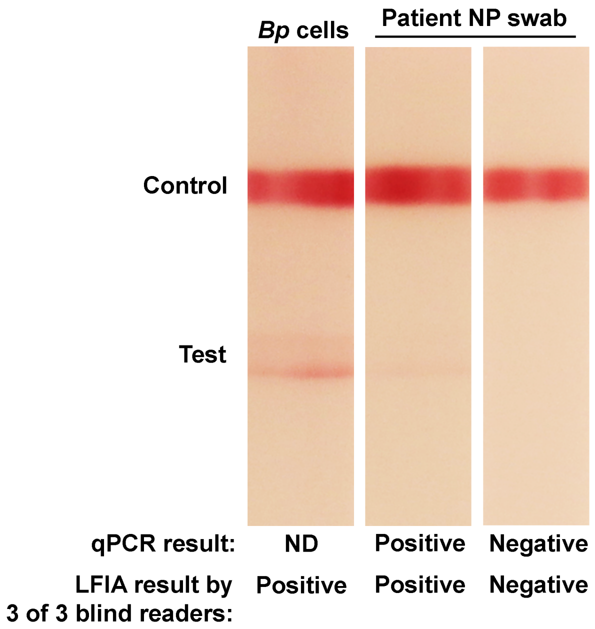

Supplementary Fig. S7. Reactivity of pertussis LFIA with patient NP swab specimens. LFIAs were tested with either *B. pertussis* (*Bp*) cells in extraction buffer or aliquots from patient NP swab specimens in Eswab Amies buffer, diluted 2-fold in PBS containing 0.5% SDS. LFIA interpretation by three of three blinded readers (*i.e.* all readers interpreted the LFIA as positive or all readers interpreted the LFIA as negative) and patient diagnosis by qPCR are indicated. ND, not determined. Note: Photography is less sensitive for interpreting weakly positive test lines than in-person visual reading of LFIAs.
